# Supplementary material for: Polygenic selection to a changing optimum under self–fertilisation
Source: PLoS Genet. 2024 Jul 17;20(7):e1011312. doi: 10.1371/journal.pgen.1011312 (PMC11285946; doi:10.1371/journal.pgen.1011312)
Supplement: S5 File — PDF printout of S2 File. (PDF) [file pgen.1011312.s005.pdf]

Polygenic selection to a changing optimum under self–fertilisation

S2 File: Dynamics at a locus underlying a quantitative trait under partial selfing

Matthew Hartfield and Sylvain Glémin

In this notebook we analyse the dynamics of a single (focal) locus underlying a quantitative trait under partial selfing.

General model

We consider a quantitative trait affected by n bi-allelic loci with additive effect:
$A_i\,A_i$: $2\,\alpha_i$
$A_i\,a_i$: α_i
$a_i\,a_i$: 0
We note x_i the frequency of allele A_i

The mean phenotypic effect of locus i is $2\,\alpha_i\,x_i$

In[∘]:=
```
Simplify[2 αi (xi^2 + F xi (1 - xi)) + αi 2 xi (1 - xi) (1 - F)]
```

Out[∘]=

$2\,x_i\,\alpha_i$

We also note z the mean phenotype of the population with $z = \Sigma_i\,2\,\alpha_i\,x_i$

In what follows we consider a single focal locus and the n - 1 other loci are considered as the back-ground.
The mean phenotypic effect of the background is thus $z_i = z - 2\,\alpha_i\,x_i$
If we neglect associations, the background is the same for the two alleles, hence the phenotype of the three genotypes are:
$A_i\,A_i$: $2\,\alpha_i + z - 2\,\alpha_i\,x_i$
$A_i\,a_i$: $\alpha_i + z - 2\,\alpha_i\,x_i$
$a_i\,a_i$: $z - 2\,\alpha_i\,x_i$
In the more general case we can note that allele A_i is associated with the background $z_i + \beta_{A,i}$ and a_i with the background $z_i + \beta_{a,i}$
As we have $\beta_{A,i}\,x_i + \beta_{a,i}(1 - x_i) = 0$.
We can thus write:
$\beta_{A,i} = \beta_i(1 - x_i)$
$\beta_{a,i} = -\beta_i\,x_i$

So the mean phenotypic effect of the three genotypes can be written as:

$A_i\,A_i$: $2\,\alpha_i - 2\,\alpha_i\,x_i + 2\,\beta_i(1 - x_i)$

$A_i\,a_i$: $\alpha_i - 2\,\alpha_i\,x_i + \beta_i(1 - x_i) - \beta_i\,x_i$

$a_i\,a_i$: $-2\,\alpha_i\,x_i - 2\,\beta_i\,x_i$

Then we can apply the classical gaussian selection model.

We note Vs = ω^2 + Ve, with ω is the standard deviation of the gaussian fitness function and Ve the environmental variance.

In what follows we remove the subscript *i* for simplicity.

In[ ]:=
```
zAA = 2 α + (z - 2 α x) + 2 β (1 - x);
zAa = α + (z - 2 α x) + β (1 - x) - β x;
zaa = 0 + (z - 2 α x) - 2 β x;
zm = Simplify[(x^2 + F x (1 - x)) zAA + 2 x (1 - x) (1 - F) zAa + ((1 - x)^2 + F x (1 - x)) zaa];
w[z_] = ⅇ^(-(z-z0)^2/(2 Vs));
Wm = Simplify[
   (x^2 + F x (1 - x)) w[zAA] + 2 x (1 - x) (1 - F) w[zAa] + ((1 - x)^2 + F x (1 - x)) w[zaa]];
```

The change in allele frequency due to selection is given by:

In[ ]:=
```
dx = Simplify[((x^2 + F x (1 - x)) w[zAA] + x (1 - x) (1 - F) w[zAa])/Wm - x]
```

Out[ ]=

$$-x + \left(e^{-\frac{(-z+z0+(-1+2\,x)\,(\alpha+\beta))^2}{2\,Vs}}\,(-1+F)\,(-1+x)\,x + e^{-\frac{(-z+z0+2\,(-1+x)\,(\alpha+\beta))^2}{2\,Vs}}\,x\,(F+x-F\,x)\right) \Big/ \left(2\,e^{-\frac{(-z+z0+(-1+2\,x)\,(\alpha+\beta))^2}{2\,Vs}}\,(-1+F)\,(-1+x)\,x - e^{-\frac{(-z+z0+2\,x\,(\alpha+\beta))^2}{2\,Vs}}\,(-1+x)\,(1+(-1+F)\,x) + e^{-\frac{(-z+z0+2\,(-1+x)\,(\alpha+\beta))^2}{2\,Vs}}\,x\,(F+x-F\,x)\right)$$

We note $\mathcal{D}$ = z0 - z, the distance of the mean trait to the optimum

We can also express z, z0, β and α in $\sqrt{Vs}$ unit to simplify the expression.

The re-scaled parameters are written with °

In[ ]:=
```
Δx[F_, 𝒟°_, α°_, β°_, x_] = Simplify[
  dx /. z → z0 - 𝒟 /. {𝒟 → 𝒟° Sqrt[Vs], β → β° Sqrt[Vs], α → α° Sqrt[Vs]}, Vs > 0]
```

Out[ ]=

$$-x + \left(e^{-\frac{1}{2}\,(\mathcal{D}^\circ+(-1+2\,x)\,(\alpha^\circ+\beta^\circ))^2}\,(-1+F)\,(-1+x)\,x + e^{-\frac{1}{2}\,(\mathcal{D}^\circ+2\,(-1+x)\,(\alpha^\circ+\beta^\circ))^2}\,x\,(F+x-F\,x)\right) \Big/ \left(2\,e^{-\frac{1}{2}\,(\mathcal{D}^\circ+(-1+2\,x)\,(\alpha^\circ+\beta^\circ))^2}\,(-1+F)\,(-1+x)\,x - e^{-\frac{1}{2}\,(\mathcal{D}^\circ+2\,x\,(\alpha^\circ+\beta^\circ))^2}\,(-1+x)\,(1+(-1+F)\,x) + e^{-\frac{1}{2}\,(\mathcal{D}^\circ+2\,(-1+x)\,(\alpha^\circ+\beta^\circ))^2}\,x\,(F+x-F\,x)\right)$$

Equilibrium ($\mathcal{D}$ = 0)

Allele frequency

We assume weak selection, so $\alpha°$, $\beta°$ << 1. We thus obtain a simple expression for the change in allele frequency:

In[ ]:=
```
Δxsel0[F_, α°_, β°_, x_] =
  Simplify[Normal[Series[Δx[F, 0 , α° ξ, β° ξ, x], {ξ, 0, 2}]] /. ξ → 1]
```
Out[ ]=

$$-\frac{1}{2}(1+3F)\,x\left(1-3x+2x^2\right)(\alpha^\circ+\beta^\circ)^2$$

We also consider symmetrical mutation

In[ ]:=
```
Δmut[u_, x_] = Simplify[ -u x + u (1 - x)];
```

So we can get the frequency at equilibrium:

In[ ]:=
```
xeq[F_, α°_, β°_, u_] = Simplify[x /. Solve[Δxsel0[F, α°, β°, x] + Δmut[u, x] == 0, x]]
```
Out[ ]=

$$\left\{\frac{1}{2},\ \frac{\alpha^\circ+3F\alpha^\circ+\beta^\circ+3F\beta^\circ-\sqrt{1+3F}\ \sqrt{-8u+(1+3F)(\alpha^\circ+\beta^\circ)^2}}{2(1+3F)(\alpha^\circ+\beta^\circ)},\ \frac{\alpha^\circ+3F\alpha^\circ+\beta^\circ+3F\beta^\circ+\sqrt{1+3F}\ \sqrt{-8u+(1+3F)(\alpha^\circ+\beta^\circ)^2}}{2(1+3F)(\alpha^\circ+\beta^\circ)}\right\}$$

And for low mutation rate

In[ ]:=
```
Simplify[Series[xeq[F, α°, β°, u], {u, 0, 1}], {α° + β° > 0}]
Simplify[Series[xeq[F, α°, β°, u], {u, 0, 1}], {α° + β° < 0}]
```
Out[ ]=

$$\left\{\frac{1}{2},\ \frac{2u}{(1+3F)(\alpha^\circ+\beta^\circ)^2}+O[u]^2,\ 1-\frac{2u}{(1+3F)(\alpha^\circ+\beta^\circ)^2}+O[u]^2\right\}$$

Out[ ]=

$$\left\{\frac{1}{2},\ 1-\frac{2u}{(1+3F)(\alpha^\circ+\beta^\circ)^2}+O[u]^2,\ \frac{2u}{(1+3F)(\alpha^\circ+\beta^\circ)^2}+O[u]^2\right\}$$

Which we can write with the initial, unscaled, parameters.

In[ ]:=
```
FullSimplify[{2 u / ((1 + 3 F) (α° + β°)^2), 1 - 2 u / ((1 + 3 F) (α° + β°)^2)} /.
   {α° → α / Sqrt[Vs], β° → β / Sqrt[Vs]}, Vs > 0]
```
Out[ ]=

$$\left\{\frac{2u\,Vs}{(1+3F)(\alpha+\beta)^2},\ 1-\frac{2u\,Vs}{(1+3F)(\alpha+\beta)^2}\right\}$$

There are two symmetrical equilibria, close to 0 or 1, and one central equilibrium at 1/2. The stability of equilibria depends on the sign of the derivative of Δx:

In[ ]:=
```
FullSimplify[D[Δxsel0[F, α°, β°, x] + Δmut[u, x], x] /. x → xeq[F, α°, β°, u]]
```
Out[ ]=

$$\left\{-2u+\frac{1}{4}(1+3F)(\alpha^\circ+\beta^\circ)^2,\ 4u-\frac{1}{2}(1+3F)(\alpha^\circ+\beta^\circ)^2,\ 4u-\frac{1}{2}(1+3F)(\alpha^\circ+\beta^\circ)^2\right\}$$

The 1/2 equilibrium is stable only if selection is weak compare to mutation, that is:

$$(\beta^\circ+\alpha^\circ)^2<\frac{8u}{1+3F}$$

Or with initial parameters

$$(\beta+\alpha)^2<\frac{8u\,Vs}{1+3F}$$

When $\beta = 0$ we retrieve the result of [1].
As stabilizing selection generates negative LD (see also the two-locus model), α and β have opposite sign, so genetic associations leads to higher equilibrium frequencies than predicted by single locus theory.

Variance components

Derivations

We can also obtain the different variance component at equilibrium.
According to [2], the total genetic variance can be decomposed into:
- The genic variance, V_g, corresponding to Hardy–Weinberg expectations and no linkage
- The inbreeding variance, V_I, corresponding to the departure from Hardy-Weinberg expectations at each locus
- The linkage covariance, C_{LD}, which takes interactions among loci into account

The genic and inbreeding variance are readily obtained from the sum of each locus contribution:

```
In[ ]:= Vg[F_, α_, β_, x_] = Simplify[x^2 (2 α)^2 + 2 x (1 - x) (α)^2 + (1 - x)^2 (0)^2]
        VI[F_, α_, β_, x_] =
         Simplify[(F x (1 - x)) (2 α)^2 + -2 F x (1 - x) (α)^2 + (F x (1 - x)) (0)^2]
```

Out[ ]=

$$2\,x\,(1+x)\,\alpha^2$$

Out[ ]=

$$-2\,F\,(-1+x)\,x\,\alpha^2$$

Which can be evaluated at equilibrium and assuming mutation rate is small.
For low frequency equilibrium:

```
In[ ]:= Simplify[Normal[Series[Vg[F, α, β, (2 u Vs)/((1 + 3 F) (α + β)^2)], {u, 0, 1}]]]
        Simplify[Normal[Series[VI[F, α, β, (2 u Vs)/((1 + 3 F) (α + β)^2)], {u, 0, 1}]]]
```

Out[ ]=

$$\frac{4\,u\,Vs\,\alpha^2}{(1+3\,F)\,(\alpha+\beta)^2}$$

Out[ ]=

$$\frac{4\,F\,u\,Vs\,\alpha^2}{(1+3\,F)\,(\alpha+\beta)^2}$$

For intermediate frequency equilibrium (1/2):

In[•]:=
```
Simplify[Normal[Series[Vg[F, α, β, 1 / 2], {u, 0, 1}]]]
Simplify[Normal[Series[VI[F, α, β, 1 / 2], {u, 0, 1}]]]
```

Out[•]=

$$\frac{3\,\alpha^2}{2}$$

Out[•]=

$$\frac{1}{2}\,F\,(\alpha+\beta)^2$$

If there is no locus at intermediate equilibrium:

$V_g = \sum_{i=1}^{n} \frac{4\,u_i\,V_s}{1+3\,F}\,\frac{\alpha_i^2}{(\alpha_i+\beta_i)^2}$

$V_I = \sum_{i=1}^{n} \frac{4\,F\,u_i\,V_s}{1+3\,F}\,\frac{\alpha_i^2}{(\alpha_i+\beta_i)^2}$

Further assuming that mutation rates and phenotypic effects are independent (or constant mutation rate) then:

$$V_g = \frac{4\,U\,V_s}{1+3\,F}\,\frac{1}{n}\sum_{i=1}^{n}\frac{\alpha_i^2}{(\alpha_i+\beta_i)^2}$$

$$V_I = \frac{4\,F\,U\,V_s}{1+3\,F}\,\frac{1}{n}\sum_{i=1}^{n}\frac{\alpha_i^2}{(\alpha_i+\beta_i)^2}$$

The covariance due to LD can also be computed as the covariance of phenotypic effect of the locus and the background.

Adapting [2], we have:

$C_{\text{LD}} = \sum_{i=1}^{n}\sum_{j\neq i}\text{Cov}(i,\,j)\,\alpha_i\,\alpha_j$

where $\text{Cov}(i,\,j)$ is the covariance between the number of allele A at locus i and j. Note that [2] considered that alleles contribute either 0 or 1 to the trait.

Note that $\text{Cov}(i,\,j)\,\alpha_i\,\alpha_j$ can be written

as:

$$2\,\alpha_i\,f(A_i\,A_i)\left(f(A_j\,A_j \mid A_i\,A_i)\,2\,\alpha_j + f(A_j\,a_j \mid A_i\,A_i)\,\alpha_j + f(a_j\,a_j \mid A_i\,A_i)*0\right) +$$
$$\alpha_i\,f(A_i\,a_i)\left(f(A_j\,A_j \mid A_i\,a_i)\,2\,\alpha_j + f(A_j\,a_j \mid A_i\,A_i)\,\alpha_j + f(a_j\,a_j \mid A_i\,A_i)*0\right) +$$
$$0*f(a_i\,a_i)\left(f(A_j\,A_j \mid a_i\,a_i)\,2\,\alpha_j + f(A_j\,a_j \mid a_i\,a_i)\,\alpha_j + f(a_j\,a_j \mid a_i\,a_i)*0\right)$$

where $f(G_j \mid G_i)$ means the frequency of genotype G_j knowing genotype G_i. [1] expressed these quantities with a set of genetic associations, for which they seek for a set of recursion equations. With our formalism, they are encapsulated in the β parameters such that:

$2\,\beta_{A,i} = 2\,\beta_i(1-x_i) = \sum_{j\neq i}\left(f(A_j\,A_j \mid A_i\,A_i)\,2\,\alpha_j + f(A_j\,a_j \mid A_i\,A_i)\,\alpha_j + f(a_j\,a_j \mid A_i\,A_i)*0\right)$

$\beta_{A,i} + \beta_{a,i} = \beta_i(1-2\,x_i) = \sum_{j\neq i}\left(f(A_j\,A_j \mid A_i\,a_i)\,2\,\alpha_j + f(A_j\,a_j \mid A_i\,a_i)\,\alpha_j + f(a_j\,a_j \mid A_i\,a_i)*0\right)$

$2\,\beta_{a,i} = -2\,\beta_i\,x_i = \sum_{j\neq i}\left(f(A_j\,A_j \mid a_i\,a_i)\,2\,\alpha_j + f(A_j\,a_j \mid a_i\,a_i)\,\alpha_j + f(a_j\,a_j \mid a_i\,a_i)*0\right)$

So we can write:

$C_{\text{LD}} = \sum_{i=1}^{n} f(A_i\,A_i)\,4\,\alpha_i\,\beta_i(1-x_i) + f(A_i\,a_i)\,2\,\alpha_i\,\beta_i(1-2\,x_i) + f(a_i\,a_i)*0*(-2\,\beta_i\,x_i)$

For one locus:

In[•]:=
```
Cld[F_, α_, β_, x_] = Simplify[(x^2 + F x (1 - x)) (2 α) (2 β (1 - x)) +
    2 x (1 - x) (1 - F) (α) (β (1 - x) - β x) + ((1 - x)^2 + F x (1 - x)) (0) (-2 β x)]
```

Out[•]=

$$-2\,(1+F)\,(-1+x)\,x\,\alpha\,\beta$$

In[ ]:=
```
Simplify[Normal[Series[Cld[F, α, β, (2 u Vs)/((1 + 3 F) (α + β)^2)], {u, 0, 1}]]]
```

Out[ ]=
$$\frac{4\,(1+F)\,u\,Vs\,\alpha\,\beta}{(1+3\,F)\,(\alpha+\beta)^2}$$

Summing over the *n* loci:

$$C_{\mathrm{LD}} = \frac{4\,(1+F)\,U\,V_s}{1+3\,F}\,\frac{1}{n}\sum_{i=1}^{n}\frac{\alpha_i\,\beta_i}{(\alpha_i+\beta_i)^2}$$

Finally, by summing the three components we obtain the total genetic variance.
For one locus:

In[ ]:=
```
VG[F_, Vs_, α_, β_, u_] =
 FullSimplify[(4 u Vs α^2)/((1 + 3 F) (α + β)^2) + (4 F u Vs α^2)/((1 + 3 F) (α + β)^2) + (4 (1 + F) u Vs β α)/((1 + 3 F) (α + β)^2)]
```

Out[ ]=
$$\frac{4\,(1+F)\,u\,Vs\,\alpha}{(1+3\,F)\,(\alpha+\beta)}$$

And summed over all loci:

$$V_G = \frac{4\,(1+F)\,U\,V_s}{1+3\,F}\sum_{i=1}^{n}\frac{\alpha_i}{\alpha_i+\beta_i}$$

Note that because the total variance must be positive, $\frac{\alpha_i}{\alpha_i+\beta_i} > 0$. As α_i and β_i are opposite sign it implies that $|\beta_i| < |\alpha_i|$

Interpretation

The expressions for variance components are not closed but they provide useful insight on the effect of genetic associations, encapsullated in β_is, on variance decomposition.

- When $\beta_i = 0$ we retrieve the house of card expression with selfing as in [1] with $C_{\mathrm{LD}} = 0$
- As α_i and β_i are of opposite sign, associations increase the genic and inbreeding variance (because selection is reduced) and make C_{LD} more negative. The overall effect is to increase genic variance compared to no associations
- Selfing has two opposite effects: increasing purging, which reduces the genetic variance but increasing associations, which increased the genetic variance. However, we can't determine the net effect, which would require to express $\sum_{i=1}^{n}\frac{\alpha_i}{\alpha_i+\beta_i}$ as a function of *F*.

Shift to a new optimum

In this part we assume that the population is away from equilibrium. Without loss of generality we assume that d0 >0.

Change in allele frequency

As above we assume weak selection, so $\gamma°$, $\beta° << 1$. We also assume that $\mathcal{D}°<<1$, which means that

the shift to the new optimum didn’t generate a strong drop in fitness.

In[ ]:=
```
Δxsel[F_, 𝒟°_, α°_, β°_, x_] =
  FullSimplify[Normal[Series[Δx[F, 𝒟°, α° ξ, β° ξ, x], {ξ, 0, 2}]] /. ξ → 1]
```

Out[ ]=

$$\frac{1}{2}(-1+x)\,x\,(\alpha^\circ+\beta^\circ)\left(-2(1+F)\mathcal{D}^\circ-(1+3F)(-1+2x)(\alpha^\circ+\beta^\circ)+(1+3F)(-1+2x)\mathcal{D}^{\circ 2}(\alpha^\circ+\beta^\circ)\right)$$

It can be re-written as the sum of two terms. The first term corresponds to pure directional selection and the second to stabilising selection

In[ ]:=

$$\text{Simplify}\left[\frac{1}{2}(-1+x)\,x\,(\alpha^\circ+\beta^\circ)(-2\mathcal{D}^\circ(1+F))\right]+\text{Simplify}\left[\frac{1}{2}(-1+x)\,x\,(\beta^\circ+\alpha^\circ)\left(-(1+3F)(-1+2x)(\beta^\circ+\alpha^\circ)+\mathcal{D}^{\circ 2}(1+3F)(-1+2x)(\beta^\circ+\alpha^\circ)\right)\right]$$

Out[ ]=

$$-(1+F)(-1+x)\,x\,\mathcal{D}^\circ(\alpha^\circ+\beta^\circ)+\frac{1}{2}(1+3F)(-1+x)\,x\,(-1+2x)\left(-1+\mathcal{D}^{\circ 2}\right)(\alpha^\circ+\beta^\circ)^2$$

This can be rewritten with the unscaled parameters

In[ ]:=

$$\text{Simplify}\left[\frac{1}{2}(-1+x)\,x\,(\alpha^\circ+\beta^\circ)(-2\mathcal{D}^\circ(1+F))\ /.\ \left\{\alpha^\circ\to\frac{\alpha}{\text{Sqrt[Vs]}},\ \beta^\circ\to\frac{\beta}{\text{Sqrt[Vs]}},\ \mathcal{D}^\circ\to\frac{\mathcal{D}}{\text{Sqrt[Vs]}}\right\},\ \text{Vs}>0\right]+\text{Simplify}\left[\frac{1}{2}(-1+x)\,x\,(\beta^\circ+\alpha^\circ)\left(-(1+3F)(-1+2x)(\beta^\circ+\alpha^\circ)+\mathcal{D}^{\circ 2}(1+3F)(-1+2x)(\beta^\circ+\alpha^\circ)\right)\ /.\ \left\{\alpha^\circ\to\frac{\alpha}{\text{Sqrt[Vs]}},\ \beta^\circ\to\frac{\beta}{\text{Sqrt[Vs]}},\ \mathcal{D}^\circ\to\frac{\mathcal{D}}{\text{Sqrt[Vs]}}\right\},\ \text{Vs}>0\right]$$

Out[ ]=

$$-\frac{(1+F)(-1+x)\,x\,\mathcal{D}(\alpha+\beta)}{\text{Vs}}-\frac{(1+3F)(-1+x)\,x\,(-1+2x)\left(\text{Vs}-\mathcal{D}^2\right)(\alpha+\beta)^2}{2\,\text{Vs}^2}$$

If we neglect LD we obtain an expression equivalent to the one of Hayward and Sella 2022 with the additional effect of selfing

In[ ]:=
```
% /. β → 0
```

Out[ ]=

$$-\frac{(1+F)(-1+x)\,x\,\mathcal{D}\,\alpha}{\text{Vs}}-\frac{(1+3F)(-1+x)\,x\,(-1+2x)\left(\text{Vs}-\mathcal{D}^2\right)\alpha^2}{2\,\text{Vs}^2}$$

Change in the phenotype

The phenotypic change can be expressed in term of the distance to the optimum. Following Hayward and Sella 2022, we can write:

$$E[\Delta\mathcal{D}]=-\sum_{i=1}^{n}E[2\,\alpha_i\,\Delta x_i]$$
$$=-\frac{\mathcal{D}}{\text{Vs}}\sum_{i=1}^{n}\left(2\,\alpha_i^2(1+F)\,x_i(1-x_i)\ +2\,\alpha_i\,\beta_i(1+F)(1-x_i)\,x_i\right)+\frac{(\text{Vs}-\mathcal{D}^2)}{\text{Vs}^2}(1+3F)\sum_{i=1}^{n}x_i(1-x_i)\left(\frac{1}{2}-x_i\right)\alpha_i(\alpha_i+\beta_i)^2$$

The first term we recognise the expressions for the variance components. In the second term, we

need to introduce notations for third order moments

$\mu_3 = \sum_{i=1}^{n} x_i\,(1 - x_i)\,\left(\frac{1}{2} - x_i\right)\alpha_i^{\,3}$

$v_{2,1} = \sum_{i=1}^{n} x_i\,(1 - x_i)\,\left(\frac{1}{2} - x_i\right)\alpha_i^{\,2}\,\beta_i$

$v_{1,2} = \sum_{i=1}^{n} x_i\,(1 - x_i)\,\left(\frac{1}{2} - x_i\right)\alpha_i\,\beta_i^{\,2}$

This leads to the expression in the main text

$$E[\Delta\,\mathcal{D}] = -\sum_{i=1}^{n} E[2\,\alpha_i\,\Delta\,x_i]$$

$$= -\frac{\mathcal{D}}{\mathrm{Vs}}\sum_{i=1}^{n}\left((1+F)\,V_g + C_{\mathrm{LD}}\right) + \frac{\left(\mathrm{Vs} - \mathcal{D}^2\right)}{\mathrm{Vs}^2}\,(1 + 3\,F)\,(\mu_3 + 2\,v_{2,1} + v_{1,2})$$

References

[1] Abu Awad, D. and Roze, D. 2018. Effects of partial selfing on the equilibrium genetic variance, mutation load, and inbreeding depression under stabilizing selection. Evolution, 72(4): 751–769

[2] Bulmer, M. G. 1976. The effect of selection on genetic variability: a simulation study. Genet. Res., 28: 101–117
