# Supplementary material for: Polygenic selection to a changing optimum under self–fertilisation
Source: PLoS Genet. 2024 Jul 17;20(7):e1011312. doi: 10.1371/journal.pgen.1011312 (PMC11285946; doi:10.1371/journal.pgen.1011312)
Supplement: Attachment — Submitted filename: Response to Reviewers.docx [file pgen.1011312.s006.docx]

**Our responses are in bold.**

**Response to Reviewer #1**

- An important limitation of this analysis is that it makes no attempt to connect quantitatively with existing theory on very related questions. For example, Hayward and Sella (eLife 2022) analyse the response to a shift in optimum for a trait under stabilizing selection in an outcrossing population in the short run (i.e, while the trait mean is changing) and in the long run (i.e., when the trait mean is more or less at the new optimum but trait variances are equilibrating, essentially under underdominant selection at each locus), focusing on how relative contribution of different loci to selection response is explained by their effect sizes, minor allele frequencies or initial variances. While their formal analyses may be difficult to extend to selfing, it is nevertheless important to try and explain the differences in selection response between populations with different selfing rates more carefully in terms of the distribution of variances, minor allele frequencies etc. across loci, and distinguish more carefully between short-term and long-term response.

**We thank the reviewer for encouraging us to tackle this important yet tricky problem. To this end, we have opened our manuscript with a more comprehensive review of existing models in the introduction (see also responses below). Then, we added an extensive analytical analysis to shed light into how self-fertilisation affects complex adaptation dynamics. We outline both a two-locus analysis and a multi-locus analysis to investigate how self-fertilisation, and genetic associations between loci, affect the response to selection.**

**Using the two-locus analysis, we demonstrate how self-fertilisation amplifies negative linkage disequilibrium generated by stabilising selection, due to reducing the efficacy of recombination. This effect impedes selection acting on selected loci following an optimum change, but diminishes over time as alleles reach their steady-state. The multi-locus analysis shows that, in the absence of associations between loci (as caused by, e.g., selective interference) then self-fertilisation can accelerate selection towards an optimum due to reducing the effective population size. However, if associations are present then such selection will be impeded due to increased inbreeding and linkage covariance.**

**This analysis adds a major new dimension to the study by demonstrating in general terms how genetic associations and self-fertilisation interact to affect polygenic selection. However, it proved intractable to fully solve the multi-allele dynamics over time, as interaction and genetic variance terms change in a complicated manner. We hence used the existing multi-locus simulation framework to more concretely investigate how polygenic selection is realised in self-fertilising species, and relate these results to the analytical solutions.**

Other related studies are those by Abu Awad and Roze (Evolution, 2018)- which the authors cite, and the one by Clo and Opedal (Evolution, 2021), both of which consider variance components in partially selfing populations, and in the case of Clo and Opedal, how these affect short term response to directional selection. There are also the papers by Kelly (199a, 1999b) which  try to predict short-term selection response (though to truncating selection) in terms of variance components. Connecting more closely to these could help go more towards explanation than description.

**We thank the reviewer for suggesting these studies. We now cite Abu Awad and Roze (2018) extensively in our revised manuscript, especially when relating our analytical solutions to theirs, which are equivalent when no associations are present (see, for example, page 16 line 286).**

**Although we do not use the mathematical approach of Kelly (1999a, 1999b) in our analytical model, we now cite these papers in the introduction when explaining how more complicated selection responses can arise under self-fertilisation. These citations are given on page 7 lines 127–128.**

**We did not feel that Clo and Opedal (2021) was entirely relevant to our mathematical analysis, as that paper investigates the role of dominance variance, whereas we assumed purely additive effects in our analysis and simulations for simplicity. However, investigating the role of dominance effects is a key goal for future work, so we have cited this paper in our discussion (on page 43 line 744).**

- Comparison of scenarios with 1 vs. 5 selected traits: It is unclear to me to what extent one can attribute differences between results for 1 vs. 5 selected traits only to pleiotropy, given that total selective effect per locus is also different across the two scenarios.
Related to this: shouldn’t variance per trait be scaled by n rather than sqrt(n) if selective effect per locus is to be kept the same as in the univariate case (lines 582-586). It is also not clear from this description how effect sizes per locus are chosen: are these supposed to be drawn from a multinomial distribution with  mean 0, variance (0.25^2)/sqrt(n) and correlation coefficient 0? In that case, the total selective effect per locus will scale as n*(0.25^2)/sqrt(n) = sqrt(n)*(0.25^2), and will thus be higher for the pleiotropic case. This is also possibly what is responsible for the smaller number of segregating mutations in the pleiotropic case (figure 4).

Thus, I think a lot more care and back-of-the-envelope quantitative arguments are needed to understand to what extent the difference in selection response in scenarios with and without pleiotropy is due to differences in selective effect per locus in the two scenarios- this would affect both the number of segregating sites (as one sees in Fig. 4), the allele frequency distribution at each segregating site before the optimum shift and the subsequent change per site under selection.

**It turns out there was a typographical error in the methods; our apologies for this confusion. It is in fact the *standard deviation* that equals 0.25/sqrt(n), *not* the variance. Hence the selective effect per locus scales as n*variance = n*(0.25/sqrt(n))^2 = n*0.25^2/n = 0.25^2, i.e. it will be independent of n. We have corrected this error in the methods (page 45 line 803).**

**We have also added new results, showing the change in trait alleles over time following the optimum shift. These figures now clearly show the change from a polygenic to a monogenic selection regime when pleiotropy is present. These results are presented in Figure 6.**

- I am not sure anything is gained by analysing “Polygenic scores” in this context. Based on the definition (lines 678-681), the polygenic score, as computed in this paper, is just half of the average trait mean (averaged over all triats). Thus, the plots showing polygenic score as a function of time (first plot in fig 4a and fig4b) seems to just be a replica of the plots showing trait mean as a function of time (first plot in fig 1a and fig 1b) modulo a factor of ½.

**Thank you for pointing this issue out. We now agree that these measurements did not tell us anything new that could not be inferred from mean fitness, so we have removed these analyses from the revised manuscript. Instead, we have focussed on tracking allele frequencies over time (see above point).**

- I am also left puzzled by the plots showing mean mutation frequency  as a function of time (second plots in figures (third plot in figs 4a, 4b and 7a). Is this the mean frequency of the minor allele at any locus? Presumably not, since it is >0.5 in some cases in fig 7a.
Alternatively, it could be the frequency of a given allele (of the two possible alleles at a locus): why then is the mean allele frequency close to zero before the optimum shift in case of the outcrossing population? Presumably, if the trait is  at the optimum, and the optimum is at the center of the phenotypic range (z0=0), then any locus effectively experiences underdominance (because of stabilizing selection), and we expect allele frequency distributions to be more or less U-shaped with mean allele frequency close to 0.5, regardless of selfing rate. See e.g., Figure 3 in The divergence of a polygenic system subject to stabilizing selection, mutation and drift; Barton (1989) for the case of an outcrossing population.
However, this is not what one observes in the third plots in figs 4a and 4b: the mean allele frequency is close to zero in outcrossing populations and 0.25 in highly selfing populations, which is quite inexplicable. My suspicion is that 10000, i.e., 2N generations of burn-in is not enough to equilibriate the population, since underdominance is known to drastically increase equilibration times. Perhaps the highly selfing populations are closer to equilibration than the outcrossing ones, presumably because of much lower N_e at higher selfing rates. If this is the case, then the comparisons of simulations at different selfing rates are not meaningful.

**The first point (regarding definitions of ‘mean variant frequency’) is now redundant as we have removed the ‘polygenic score’ plots.**

**In this revised manuscript we have repeated the main simulations so that the burn-in is now 10N generations, as opposed to 2N. However, this longer burn-in does not affect the results, implying that sufficient time has elapsed before the optimum shift so that the population has reached a steady-state.**

**Instead, we think that the differences instead arise due to the different way mutations affect trait values in our simulations compared to other models. In Barton 1989, for example, mutations have symmetric fitness effects; that is, wild-type and derived homozygotes have an effect of -a and +a on the trait, respectively. Conversely, in our simulations, mutations instead have a directional effect; wild-type mutations have no trait effect; heterozygotes have an effect +a, derived homozygotes an effect of +2a. This directional selection, combined with the stabilising selection assumptions, means that mutations will reach mutation-selection balance. explaining the low frequency in outcrossing populations. It would be similar to take the minor allele frequency in Barton 1989 instead of the full distribution (for which the mean is indeed 0.5 because of symmetry).**

**Given this important difference in modelling assumptions, we have made this assumption more explicit in the main text by highlighting it when outlining our analytical approach (page 9 lines 179–182).**

- A related point: Mean mutation frequency and Mean effect are not very meaningful quantities to report, as the same change in mean allele frequency can be due to subtle shifts at many loci vs. substitution at a few loci. It would be more informative to show how the distribution of allele frequencies changes, e.g., by plotting the distribution before the shift along with the distribution at a few well-chosen  time points during adaptation. There can be other ways of exploring this, e.g., see Hoellinger et al (2019) or Hayward and Sella (2022), but just plotting mean allele frequency essentially obscures all patterns of interest, making it hard to judge to what extent adaptation is indeed polygenic.

**This is an excellent suggestion, and to this end we have now plotted frequency changes of the trait alleles over time. These results now clearly show how the dynamics of adaptation change differ both (i) due to the mating system, where we see more coordinated change in allele frequencies due to selfing, and (ii) due to pleiotropy, where we observe a transition towards a monogenic selection regime driven only by a few trait mutations.**

**We have presented these new results in Figure 6.**

- More care is needed in disentangling to what extent the degree of selfing affects response by affecting the magnitude of the variance components (before the optimum shift) vs. actually affecting the selection gradients. For example, why is the change in trait mean so similar across different selfing rates (fig 1a), even though variance components prior to the shift in optimum are quite different (fig2a). Connecting to previous theory: Clo & Opedal (2021); Kelly (1999a,b) may give some insight.

**This is a point where the new analytical solutions help us interpret the simulation results. By extending the model of Hayward and Sella, we show that under the ‘rapid’ phase when the population is adapting to an optimum, selfing has no effect due to the increase in both homozygosity and drift cancelling out. However, under the ‘equilibrium’ phase when an optimum is reached, the analytical results predict increased efficacy of selection. These new analytical findings help explain why selfing has a longer-term fitness advantage.**

**We explain these analytical results on page 17 lines 306–309, and their use in interpreting simulation results on page 1197 lines 345–346.**

Lines 170-175: The references here are not apt, and the interpretation is not correct: Hoellinger et al do not consider a model of stabilizing selection; de Vladar and Barton consider stabilizing selection but without drift- which can be misleading, since stable intermediate-frequency polymorphisms can be maintained in infinite populations, but not in finite populations (Nu<<1) under stabilizing selection.
In general, once the mean has reached the new optimum, variance will equilibrate to a level very close to the level before the optimum shift, and this should occur via near fixation of compensatory mutations (because of effective underdominance per locus)- regardless of selfing rate or pleiotropy. See Hayward and Sella (2022) for more details. However, this may occur over different timescales depending on pleiotropy and selfing.
More generally, the distinction between sweeps vs. shifts is relevant only for the initial rapid phase (while the trait mean is changing): one could ask, is the change in trait mean achieved via sweeps (near fixations) or subtle allele frequency shifts?  The long-term equilibration in variance is always achieved via near-fixation of alleles.

**We have decided to delete the references to the Hollinger paper here, as we agree that they could confuse readers. However, we retained the reference to de Vladar and Barton 2014 here with an extra clarification, as our goal was to reference the behaviour where variance temporarily spikes during adaptation, as observed in previous polygenic selection papers.**

**Furthermore, we further discuss the mechanism of adaptation (sweeps vs polygenic shifts) later in the manuscript, when we instead discuss the role of linkage disequilibrium and the new allele-frequency change plots. Examples of these discussions can be found on page 29 lines 470–476.**

Lines 176-180; lines 217-219: This may again not be about pleiotropy per se, but stronger selection per locus? See also “A population genetic interpretation of GWAS findings for human quantitative traits”; Simons et al (Plos Biology, 2018) for analysis of selective effect and genic variance per locus under pleiotropy.

**We refer the reviewer to the above point on how there was a typographical error in the previous version of the manuscript, which would have otherwise implied that selection did not scale with pleiotropy.**

Lines 391-393: Rescaling recombination rates by 1-F is a deterministic approximation and is not expected to work once drift and Hill-Robertson interference become important, i.e., for selfing rates > 0.9, and large U and/or with recessive alleles; see e.g., Kamran-Disfani and Agrawal.

**We did not use the simple 1–F rescaling; we instead rescaled recombination rates to take joint identity-by-descent into account (as outlined by Roze 2009), albeit in an approximate manner due to the large genome sizes simulated. This is more precise under high self-fertilisation and recombination rates. Note also that U is not large in our simulations so that the Roze’s approximation works well.**

**We have outlined this method and its implementation on page 47 lines 839–857.**

**Response to Reviewer #2**

In this paper, the authors use simulations to study polygenic adaptation with varying selfing rate.  They start the simulations with stabilizing selection to a fixed optimum and then shift the optimum (either for one trait or a vector of traits).  The main result is negative: the rate of selfing seems to have no effect on the change in mean phenotype(s) in response to environmental change.  There are some effects of selfing rate on the genetic variance (e.g. fixation of variants with compensatory effects in elevated linkage disequilibrium).  In general, the authors present a thorough treatment of an important problem.

I have two major suggestions for a revised ms.  First, in its current form, the paper will not engage the broadest audience since it is focused on technical details related to mating system.  I suggest that the main negative result – selfing rate does not matter for adaption to a novel environment – should be presented as contrary to the popular wisdom.  The idea that selfing taxa are short lived (Line 73 onward) is usually attributed to the limited evolutionary potential of selfers.  This study undermines that belief.  Related to this angle, the ‘burn-in’ period of the simulations merits greater discussion – Is there actually less genetic variation available to selection in highly selfing populations at the time of environmental change?  How does this aspect of the model match up to empirical data on standing levels of quantitative trait variation within selfing and outcrossing species?

**We thank the reviewer for their positive comments on the manuscript. While we agree that it is important to consider how these results affect the long–term fitness of selfing lineages, we feel that it is not the sole message of the manuscript. Following revisions, it has now become a more comprehensive analysis of how polygenic adaptation is realised in selfing species across different timescales (i.e., what the short-term effects are compared to the longer-term ones), and how these changes manifest themselves in genome data. The extra mathematical analytics and comments from Reviewer 1 have further broadened the scope of this manuscript to consider the theoretical basis of these adaptive changes. As a conclusion, we feel it is too simplistic to say that selfing ‘does not matter’; it has many interacting effects on adaptation, including benefits once an optimum has been reached. For these reasons, we do not feel it is best to primarily focus solely on the adaptation rate over the short-term.**

**With regards to genetic variance, we have now added new analytical equations showing how genetic variance is partitioned in selfers, and how selection interference affects these measurements. See Equation 12 in the revised manuscript. We are hence better able to discuss how genetic variance is affected due to the mating system and selection interference; in particular, increased interference increases additive genetic variance but also causes more negative LD covariance. We discuss these results in relation to our simulations on page 21 lines 374–375.**

**Finally, our results show that, even though selfing species can exhibit lower levels of trait variation, this does not limit their ability to adapt to new environments. This finding is in line with previous theoretical results and an empirical meta-analysis by Clo *et al.* 2019, which showed how non-additive variance components can contribute to trait heritability, and hence a selection response. We have added this point on page 43 lines 751–754 in the discussion.**

Second, I am struggling a bit to understand the significance of the results on the genetic variance.  For example (lines 361-364): “we observe that once the optimum has been reached, highly selfing populations exhibit the highest fitness and lowest inbreeding depression (Figure 1). This advantage likely arises as these populations also exhibit the lowest variance in fitness through purging maladaptive genotypes…”  If I understand the simulation however, a population ends up in the same scenario where it started.  Each population starts at equilibrium between mutation, drift, and stabilizing selection with a fixed optimum.  Then the optimum shifts and the population evolves until it reaches the new optimum.  Then the variance will settle into an equilibrium of mutation, drift, and stabilizing selection around the new optimum.  But the location of the optimum does not affect the equilibrium (co)variances, right?  So are these variance affects transient OR were they features of the selfing population before the optimum changed?

**With regards to ‘macroscopic’ level effects (e.g., mean fitness, genetic variance), the new equilibrium is reached rapidly so these effects can be transient; indeed, this is what is observed under some selfing cases when there is a temporary spike in variance as adaptation is ongoing. However, the genetic structure can have long-lasting effects; in particular, the underlying block-like structure can persist for a long period of time in selfers.**

**We have added a sentence to the discussion on this point, on page 38 lines 636–638.**

Minor comments:

Line 4 and elsewhere: Replace “underlying variants” with loci.

**We prefer to retain the phrase ‘variants’ in our manuscript, as this seems to be the commonly–used terminology in complex-trait genetics. This term also allows us to focus on how these alleles change in frequency over time. In contrast, ‘loci’ usually refers to the actual sites rather than the variants within; we hence feel that making this change would risk confusing readers.**

Line 57: Is “sex–cells” different from gametes?

**This is correct; we have now made this change to the manuscript.**

Lines 134-136: “We used the SLiM software (Haller and Messer 2019) to simulate a large population (N = 5000) where each individual consisted of a 25Mb chromosome consisting of alternating neutral and gene regions”.  One always establishes a “base parameter set” with forward simulations but the reader needs assurance that arbitrary choices do not matter.  Adding some text like “We performed a small number of simulation with more than one chromosome and a population size of N=20000 and found equivalent results…” would be good here.

**We have replicated basic results (i.e., without pleiotropy or deleterious mutations) but with a larger population size of N = 10,000 (we kept the burn-in the same length due to time and memory constraints). We find the same qualitative results as with a smaller population size.**

**We have mentioned the inclusion of these results on page 31 lines 484–487 (and in S3 File), and also in the Methods section on pages 46–47 lines 834–838.**
